# Respiratory Syncytial Virus G Protein Sequence Variability among Isolates from St. Petersburg, Russia, during the 2013–2014 Epidemic Season

**DOI:** 10.3390/v13010119

**Published:** 2021-01-17

**Authors:** Vera Krivitskaya, Kseniya Komissarova, Maria Pisareva, Maria Sverlova, Artem Fadeev, Ekaterina Petrova, Veronika Timonina, Anna Sominina, Daria Danilenko

**Affiliations:** 1 Department of Etiology and Epidemiology, Smorodintsev Research Institute of Influenza, 197376 Saint-Petersburg, Russia; vera.krivitskaya@influenza.spb.ru (V.K.); maria.pisareva@influenza.spb.ru (M.P.); maria.sverlova@influenza.spb.ru (M.S.); artem.fadeev@influenza.spb.ru (A.F.); polyanskayaekaterina@yandex.ru (E.P.); anna.sominina@influenza.spb.ru (A.S.); daria.danilenko@influenza.spb.ru (D.D.); 2 Children’s City Hospital of St. Olga, 194017 Saint-Petersburg, Russia; karina88888888@list.ru

**Keywords:** human respiratory syncytial virus, phylogenetic analysis, ON1/GA2 genotype, G protein variability

## Abstract

Human respiratory syncytial virus (RSV) is the most common cause of upper and lower respiratory tract infections in infants and young children. It is actively evolving under environmental and herd immunity influences. This work presents, for the first time, sequence variability analysis of RSV G gene and G protein using St. Petersburg (Russia) isolates. Viruses were isolated in a cell culture from the clinical samples of 61 children hospitalized (January–April 2014) with laboratory-confirmed RSV infection. Real-time RT-PCR data showed that 56 isolates (91.8%) belonged to RSV-A and 5 isolates (8.2%) belonged to RSV-B. The G genes were sequenced for 27 RSV-A isolates and all of them belonged to genotype ON1/GA2. Of these RSV-A, 77.8% belonged to the ON1(1.1) genetic sub-cluster, and 14.8% belonged to the ON1(1.2) sub-cluster. The ON1(1.3) sub-cluster constituted a minor group (3.7%). Many single-amino acid substitutions were identified in the G proteins of St. Petersburg isolates, compared with the Canadian ON1/GA2 reference virus (ON67-1210A). Most of the amino acid replacements were found in immunodominant B- and T-cell antigenic determinants of G protein. These may affect the antigenic characteristics of RSV and influence the host antiviral immune response to currently circulating viruses.

## 1. Introduction

Human respiratory syncytial virus (RSV) is the most common cause of respiratory tract infections in infants and young children worldwide and a major public health burden [1,2]. In developed countries, the prevalence of RSV infection reaches 42–63% in children under 3 years of age hospitalized with acute lower respiratory tract illness. In children, RSV is implicated in: 50–90% of bronchiolitis; 5–40% of pneumonia; and 10–30% of tracheobronchitis [3,4,5]. During the 2012–2013 epidemic season in St. Petersburg, the frequency of RSV infection was 13.5% (comparable to that of influenza) among hospitalized children in the first year of life [6].

RSV features a negative-sense, unsegmented RNA genome and belongs to the family *Pneumoviridae*, genus *Orthopneumovirus*. Its envelope consists of two transmembrane glycoproteins: fusion protein (F) and attachment protein (G) that interact with the receptors on the cell surface. Human RSV exists as a single serotype, but is divided into two antigenic groups (RSV-A and RSV-B), depending on interactions with monoclonal antibodies [7].

The molecular epidemiology of RSV is based mainly on G gene phylogeny. According to the G gene sequence, human RSV-A is divided into 12 genotypes (GA 1-7, SAA1, NA1, NA2, ON1, ON2); RSV-B is divided into 21 genotypes (GB 1-4, BA 1-11, SAB 1-4, and URU 1-2) [8,9,10]. RSV-A and RSV-B co-circulate, in various proportions, during almost all epidemics. In most cases, RSV-A dominates [8,11,12,13].

The pathogenesis of RSV infection is complex and often ambiguous. Depending on the circumstances, RSV can induce a protective, Th1-dependent immune response or a Th2-mediated immunopathology (bronchial hyperreactivity and obstruction, pulmonary eosinophilia) [14]. Numerous studies have established the roles of certain G protein antigenic determinants in both antiviral protection and immunopathology [15,16]. Changes in certain G protein epitopes can significantly affect viral properties as a whole; these may enable evasion of an immune response, thereby resulting in frequent reinfections [17,18,19].

RSV is actively evolving under the influence of environmental and herd immunity factors. Understanding this pathogen’s evolutionary patterns necessitates the monitoring of the genetic and antigenic variability of viruses in human circulation from different geographic locations. Furthermore, the study of RSV evolution is crucial for vaccine and antiviral drug development. The aim of this study was to identify the types of RSV circulating in St. Petersburg during the 2013–2014 epidemic season and to evaluate the variability of the RSV-A G protein.

The present study confirms previous findings that the RSV-A viruses of the ON1 genotype superseded all the other genotypes. The genetic diversity of RSV isolates from Russia was analyzed with a special focus on the non-synonymous mutations in immunodominant B- and T-cell epitope-coding regions of the G gene.

## 2. Materials and Methods

### 2.1. Clinical Samples

Nasopharyngeal swabs were obtained from children (from 2 weeks to 6 years with median age 12.9 mon. (±11.4 mon.)) within the framework of a GIHSN study at the Smorodintsev Research Institute of Influenza (RII) from January to April 2014. All research procedures involving humans were performed in strict accordance with the ethical standards of the committees (local ethical committee and national standards), and with the Helsinki Declaration of 1975, as revised in 2008. The study protocol was approved by the local RII ethics committee (registered under No. 0005131, 2008, International IRBS for Russia). The parents of all patients provided written informed consent.

### 2.2. Isolation of RSV in Cell Culture

Isolation of RSV from PCR-confirmed clinical samples was carried out in MA-104 cell culture (obtained from the Cell Culture Collection of the Institute of Cytology, Russian Academy of Sciences, St. Petersburg, Russia) using alpha-MEM serum-free support medium (BioloT, Saint Petersburg, Russia).

### 2.3. RT-PCR of the G Protein Gene

RNA was extracted from MA-104 cell culture supernatants (containing primary passages of RSV isolates) using the QIAampViral RNA Mini Kit (QIAGEN, Dusseldorf, Germany). The One-Step RT-PCR kit (QIAGEN, Dusseldorf, Germany) was used to amplify the G gene open reading frame. An in-house system was used for subtyping RSV-A and RSV-B. M13 sequences were added to products’ 5′ ends during amplification of the RSV-A G gene (Table 1).

### 2.4. Capillary Sequencing

PCR products were then extracted from RT-PCR reaction mixtures using the QIAquick PCR purification Kit (QIAGEN, Germany). Sequencing was performed using an ABI GA 3130 (Applied Biosystems, Foster City, CA, USA) and the BigDye Terminator v3.1 Cycle Sequencing Kit (ThermoFisher Scientific, Waltham, MA, USA).

### 2.5. Phylogenetic Analysis of the G Gene (Nucleic Acid) Sequences

For sequencing, primary passages of 26 RSV-A strains isolated in cell culture were used. G protein gene sequences were aligned with available RSV-A sequences (from GenBank) using the MUSCLE algorithm included in MEGA 7. A maximum-likelihood phylogenetic tree was constructed using MEGA 7 (TN93+G, bootstrap 1000 replications). G gene sequences of strains representing each known RSV-A genotype were retrieved from GenBank and included in the tree (Table 2). High-throughput phylogenetic analysis of the GenBank RSV-A dataset was performed using RAxML [20]. A tree was visualized using the R ‘ggtree’ package [21]. Genetic distances were calculated using dist.alignment function in the R ‘seqinr’ package [22].

### 2.6. RSV Glycosylation Site Analysis

Putative N-glycosylation sites were predicted using the *NetNGlyc 1.0 server (threshold ≥ 0.5).

O-glycosylation sites were evaluated using the **NetOGlyc 4.0 server (G-score ≥ 0.5).

### 2.7. Statistical Analysis

The Pearson association coefficient (rA) was used to estimate the relationship between the presence of complications and RSV antigenic groups in the analysis of the contingency table.

## 3. Results

RSV were isolated in cell culture from the clinical samples of 61 children hospitalized (January–April 2014) with laboratory-confirmed RSV infection. Patient ages ranged from 2 weeks to 6 years. According to RT-PCR data, 56 isolates (91.8%) belonged to RSV-A, and only 5 (8.2%)—RSV-B. A complicated course of RSV infection was observed in 14 out of 61 individuals (23.0%) with ages from 6 months to 3 years (median age 9.4 month ± 3.1). Of those 14, the following complications were recorded in patients with RSV-A: nine acute bronchitis cases (64.3%); one bronchiolitis case (7.1%); two otitis media cases (14.3%); and one acute pneumonia case (7.1%). With RSV-B, one case of acute bronchitis (7.1%) was noted.

The RSV G gene was sequenced for 26 RSV-A strains. All of these viruses belonged to the ON1 genotype (ON1/GA2, according to the latest classification revision [23]). Currently, ON1/GA2 viruses are ubiquitously dominant among RSV-A circulating globally [10,13,23,24,25,26,27,28,29].

The genetic diversity of Russian RSV isolates corresponds to the observed genetic diversity of RSV sampled in other regions (Supplementary Figure S1).

**Figure 1:**
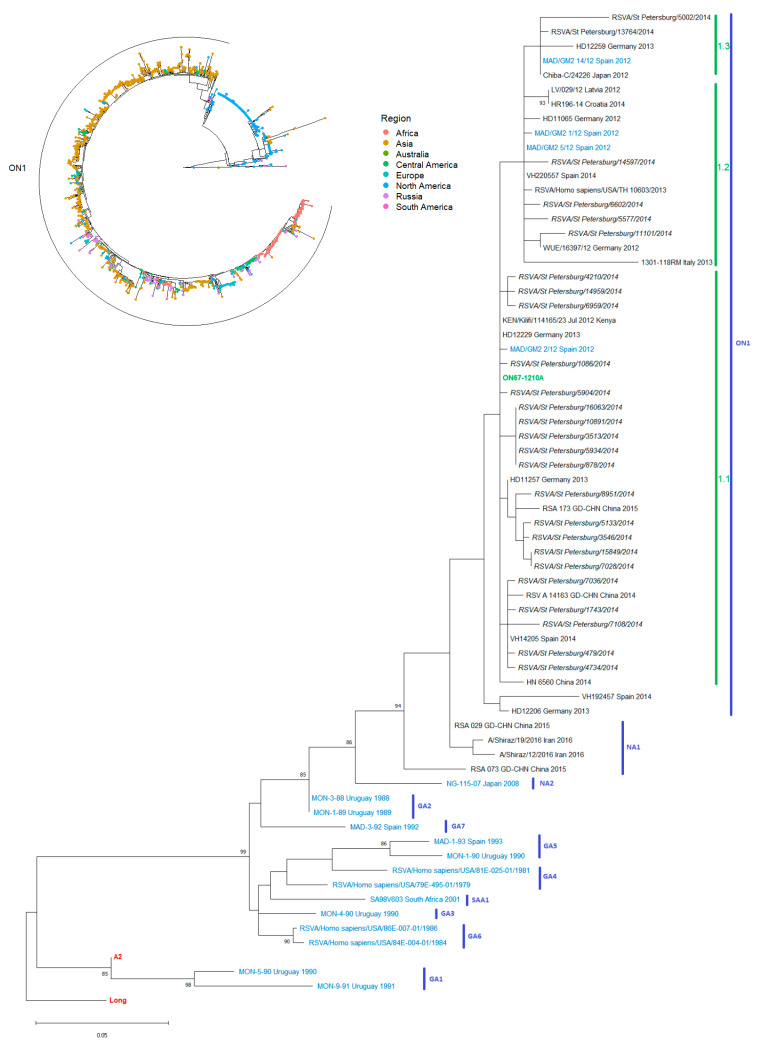
Phylogenetic trees by G gene sequence, St. Petersburg RSV-A isolates, 2013–2014 epidemic season. Radial tree (1491 sequences from GenBank) with a focus on geographic distribution. Rectangular tree with reference strains for each genotype colored in blue; original Canadian reference virus (ON67-1210A)—green; progenitors of RSV-A (Long and A2 strains) colored in red.

All of the viruses analyzed were genetically close to the reference (ON1/GA2 strain ON67-1210A) (Figure 1). According to the Tabatabai classification [11], division of the RSV-A ON1/GA2 genotype into sub-clusters is based on amino acid substitutions at certain positions (274, 298, 303, 304, 310), compared to the G protein of the reference ON67-1210A. Viruses that are as close as possible to the reference virus are classified as sub-cluster ON1(1.1). Sub-cluster ON1(1.2) is characterized by mutations L274P, L298P, and Y304H. The ON1(1.3) viruses contain V303A and L310P substitutions.

Most of the isolates (21/27 strains, 77.8%) belonged to the ON1(1.1) sub-cluster. Four strains (14.8%) could be identified as ON1(1.2). ON1(1.3) constituted a minor group: only two viruses (7.4%), RSVA/St_Petersburg/5002/2014 and RSVA/St_Petersburg/13764/2014, had substitutions in all five critical positions (L274P, L298P, V303A, Y304H, L310P) (Figure S2).

**Figure 2:**
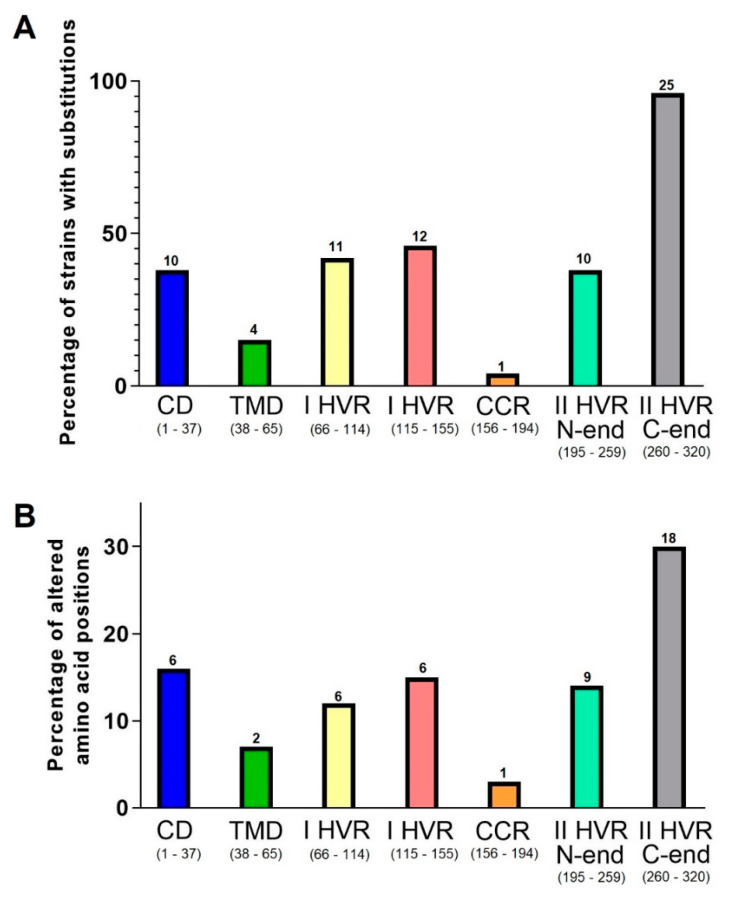

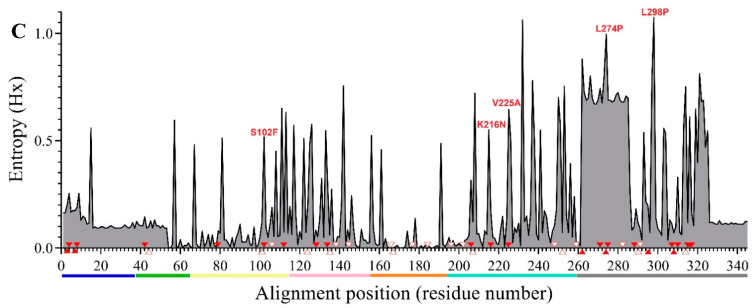
Variability of G protein structural regions compared with the reference strain. Comparison uses St. Petersburg RSV-A isolates (n = 27) from the 2013–2014 epidemic season. The rate of strains (**A**) and position frequencies (**B**) with aa substitutions identified in specific G protein structural domains. In brackets, in the signature to the X-axis, the numbering and quantity (n) of the amino acid residues of the regions are indicated. Above the diagrams, the number of divergent strains (**A**) or amino acid positions (**B**) is indicated. The positions of the amino acid substitutions are shown with red triangles (filled and empty for non-unique and unique mutations, respectively) at the Shannon entropy plot (**C**) (based on 1491 sequences available in the GenBank database). CD—central domain, TMD—transmembrane domain, I HVR—I hypervariable region, II HVR—II hypervariable region.

Four strains had substitutions at two positions in the transmembrane region: T42I (RSVA/St_Petersburg/479/2014, RSVA/St_Petersburg/7108/2014, RSVA/St_Petersburg/6021/2014) and S44A (RSVA/St_Petersburg/5002/2014). In two strains, there are several substitutions in the central domain: RSVA/St_Petersburg/4734/2014 (V167G, F168V, S177R) and RSVA/St_Petersburg/6959/2014 (A184S). In 11 strains, we identified substitutions in 6 positions in the cytoplasmic domains. Nineteen St. Petersburg strains had point mutations at 12 positions in the first hypervariable region, and all studied strains have aa substituted in the second hypervariable region. There are also six positions with substitutions in the heparin-binding domain (Figure 2; Supplementary Table S1).

Using the program NetOglyc, 33 and 48 O-bond Ser and/or Thr were predicted as potential O-glycosylation sites in I and II hypervariable regions of the ON67-1210A virus with a high likelihood: the Matthews correlation coefficient (MCC) was 0.9040–0.9920. According to NetNGlyc, the following N-glycosylation sites were identified for the ON67-1210A virus: two potential sites with scores > 0.5 in the first hypervariable region (103NLS105, 135NTT137) and one in the second hypervariable region (237NTT239). Compared with the ON67-1210A strain, O-glycosylation sites were highly variable in St. Petersburg viruses, while N-glycosylation sites were quite conserved.

In 13 of 26 isolates (48.1%), one or two substitutions were found, at six positions in the first hypervariable region, which result in the loss of O-glycosylation sites (S100G, S102F, T112I, S124P, S128F, T138I). In only one strain (RSVA/St_Petersburg/8951/2014), a substitution (N135D) leading to the loss of an N-glycosylation site was observed in addition to those causing loss of two O-glycosylation sites (S100G and S102F). In 15 out of 26 isolates (55.6%), a loss of one or two potential O-glycosylation sites was detected at nine positions in the second hypervariable region (T207I, T259K, S275G, T282A, T288A/N, S291P, S315P, S316P/F, S317C/A). Almost all G protein glycosylation pattern changes occurred at B cell antigenic determinants previously identified in animals (aa 90–110, 129–152, 169–207, 236–263, 263–298) [19] and humans (aa 90–168, 169–207, 236–298, 262–276) [17,30].

## 4. Discussion

An association between the clinical manifestations of infection and genetic traits of currently circulating RSV has been demonstrated [12,13]. However, we did not observe differences in the incidence of complications in patients infected with RSV-A or RSV-B (rA = 0.02, *p* > 0.5). This may be due to the insufficient size of the RSV-B data.

Despite the relative genetic homogeneity of viruses from St. Petersburg, multiple single-amino acid changes were identified in the G protein, compared with the ON67-1210A strain. It has been shown that changes in RSV G protein antigenic determinants can significantly affect viral properties and help it escape immune response [17,18,19].

In four strains, we find substitutions at two positions in the transmembrane region: T42I and S44A. There is little information available on the variability of the transmembrane domain. However, several changes in this region (S51P, L54P) reduced the affinity of RSV-specific neutralizing antibodies [31].

The central domain of the G protein is involved in interactions with host target cells [18,32], and this may explain its high level of sequence conservation. Only in one strain were substitutions detected in this region: RSVA/St_Petersburg/6959/2014 (A184S). This substitution is of special interest. It is located in the important, regulatory T cell epitope (aa 183–195) that stimulates in vitro production of both Th1-mediated (interferon-ɣ, IL-2) and Th2-mediated (IL-4, IL-5, IL-6, IL-13) cytokines in splenocytes. Depending on experimental conditions, mice pre-immunized with a synthetic peptide corresponding to this epitope develop either Th1-dependent protection or Th2-mediated immunopathology (lung eosinophilia) during subsequent RSV infection [33,34]. In addition, position 184 is part of an RSV-A G protein site (182CWAIC186) that mimics part (aa 202–207) of the fractalkine (the member of the CX3C chemokine family). RSV interacts with the fractalkine receptor CX3CR1 expressed on immunocompetent cells. The synthetic peptide 182–186 acts as a fractalkine antagonist blocking CX3CR1 in RSV-infected mice. This leads to inhibition of the influx of CX3CR1+ inflammatory cells to sites of infection. As a result, a decrease in the RSV-specific protective immune response was observed [35,36].

Surprisingly, the cytoplasmic domains of the investigated viruses had a significant degree of variability. In nine strains (33.3%), five positions showed variation of 1–2 aa residues (T4P/N, K5Q/H, Q7P, R8H, K11R). The sequence variability of this region is also observed in the large GenBank dataset (see Figure 2C) and supported by sequences obtained by using different technologies. Currently, there is no literature data about substitutions in this region, as identified in our isolates. Despite the fact that this region is not exposed on the cell surface, it contains B cell epitopes, mutations in which can affect G protein’s antigenic characteristics. For instance, mutations L33P and L35P led to RSV-A being unable to interact with neutralizing monoclonal antibodies [31].

Few studies are devoted to researching the variations in the first hypervariable region of circulating RSV. In comparison with the ON67-1210A virus, 19 St. Petersburg strains (70.4%) had point mutations at 12 positions in this domain. Altered aa position frequencies were found to be distributed almost evenly at the N- and C-termini of this domain. In 68.4% of mutant strains (13/19), substitutions were identified directly in known B cell epitopes (aa 66–90, 90–110, 129–152) [19] and CD4+ T cell epitopes (aa 104–118) [33]. For instance, the S102F substitution (at the previously evolutionarily conserved position) was first noted as typical for ON1/GA2 viruses circulating in the Netherlands in 2016/2017 [13]. That mutation was detected in five of our viruses, yet was isolated in 2014.

The second hypervariable region is G protein’s most changeable domain. In comparison with the ON67-1210A virus, 100% of the St. Petersburg strains had substitutions in this domain. It was found that 27 positions were variable and asymmetrically distributed. The identified changes were observed two times more often at the C-terminus than at the N-terminus of this region. The same applies to the frequency of strains with substitutions at a different part of the G protein amino acid sequence.

In our isolates, most changes in this domain were found in CD4+ T cell determinants (aa 193–203, 208–222) [37,38] and in B cell immunodominant determinants of both animals (aa 169–207, 236–263, 245–274, 263–298) [19] and humans (aa 229–240, 236–298, 265–273, 283–291) [17,30]. It has been found that some of the substitutions identified in seven of our strains (P206Q, T207I, V225A, L274P, or L310P) can affect the anti-RSV immune response [17,31,39]. It should be noted that the single strain (RSVA/St_Petersburg/11101/2014) that had combined mutations at two positions (V225A, L274P) was isolated from a child, aged 22 days, with severe complicated laboratory-confirmed RSV infection.

In 10 isolates (37.0%), one or two mutations were identified at six positions (K196M, P202L, P206Q, T207I, K216N, V225A/L) in the heparin-binding domain (aa residues 187–231), which can act as a viral receptor. This region is rich in positively charged aa residues and seemed a likely candidate for interaction with negatively charged cell surface glycosaminoglycan-heparan sulfate [40]. Moreover, these positions are part of an epitope (aa 177–220) which can contribute to immunopathological manifestations (lung eosinophilia) in infected mice [41].

Twelve positions, in which substitutions were identified in our strains, have been previously shown to be positively selected in ON1/GA2 viruses [10,12,24,42]. In our case, these are: V225A, L248I, E271K, L274P/V, S275G, H290Q, L298P, V303A, Y304H, L310P, L314I, and S317C/A. This suggests that they are important for the development of antiviral immunity, and their variability may be shaped by host immune pressure.

About 60% of the RSV G protein is glycosylated. The high content of glycans prevents effective antigenic presentation of the G protein to immunocompetent cells and allows RSV to avoid the host immune response [43,44,45].

At some RSV-A G protein positions, the reverse amino acid substitutions have been observed several times during viral evolution [10,31,46]. Such replacements have been termed “flip-flops”. When compared with the ON67-1210A strain, 2 known “flip-flop” substitutions were identified in 6 of 26 (22.2%) St. Petersburg isolates: V225A (V↔A); and/or L274P (L↔P). Those positions (225 and 274) have been reported to be under positive selection in ON1/GA2 viruses; this strongly suggests that their variations reflect changes in the immune status of human populations and are the result of immune pressure [10]. Changes at 225 and 274 have been associated with the decreased affinity of specific neutralizing antibodies [31].

Interestingly, our isolates either did not have substitutions at position 274, or had combined substitutions: L274P and L298P (Figure 2). Thus, these isolates had the haplotype PP at positions 274/298 in contrast with the reference strain (ON67-1210A) with a haplotype LL. For the RSV-A ON1/GA2 genotype, aa residue 298 is part of an insertion (aa residues 284–307) that appeared as a duplication of aa 261–283 [42]. As a result of duplication, aa residue 274 corresponds to aa residue 298. Presumably, substitution at position 298, like at 274, may also be reversible in a “flip-flop” (L↔P) manner. This is supported by RSV (ON1/GA2) G protein structures seen in isolates from 2012 to 2017 in many regions of the world. In addition to viruses with the parent LL haplotype and/or modified PP haplotype at positions 274/298, RSV-A viruses featuring a mixed (LP and/or PL) haplotype have been identified in Kenya, Germany, China, Thailand, Bulgaria, the USA, and the Netherlands [11,13,24,25,26,27,47].

## 5. Conclusions

The data collected and presented in this study show that, during the 2013–2014 epidemic season in St. Petersburg, mainly RSV-A viruses were circulating. According to RSV-A G gene sequence analysis, isolates are close to the ON67-1210A reference strain and belong to the ON1/GA2 genotype, without formation of a separate clade. However, multiple point mutations, identified in antigenic immunodominant B- and T-cell epitopes of the G protein, may be the result of immune pressure and were able to affect the antigenic characteristics of currently circulating RSV-A. The wide variability of surface RSV glycoprotein, responsible for the induction of protective antiviral reactions, may contribute to the emergence of RSV variants with a potential to escape immune response.

## Figures and Tables

**Table 1:** Primers used for amplification of the RSV-A G gene.

Primer	Sequence
M13F-hRSVAB-G-F	5′-TGTAAAACGACGGCCAGTGCAAATGCAAACATGTCCAAA-3′
M13R-hRSVA-G-R	5′-CAGGAAACAGCTATGCAACYATACGCTTTTTAAATGACTA-3′

**Table 2:** RSV-A G protein’ gene GenBank sequences.

Strain	GenBank Accession No.	Strain	GenBank Accession No.
ON67-1210A	JN257693	MON-1-89	Z33422
ATCC VR-26 (Long)	AY911262	MON-5-90	Z33427
A2	KT992094	MON-1-90	Z33494
RSVA/Homo_sapiens/USA/79E-495-01/1979	KP856969	MON-4-90	Z33426
RSVA/Homo_sapiens/USA/81E-025-01/1981	KP856967	MON-9-91	Z33431
RSVA/Homo_sapiens/USA/84E-004-01/1984	KP258733	MAD-3-92	Z33455
RSVA/Homo_sapiens/USA/86E-007-01/1986	KP258723	VH220557	KM402663
MAD-1-93	Z33414	HN_6560	KT781349
MAD/GM2_14/12	KP792373	RSV_A_14163_GD-CHN_2014	KX009698
MAD/GM2_1/12	KP792361	RSA_073_GD-CHN_2015	KX009700
MAD/GM2_2/12	KP792362	RSA_029_GD-CHN_2015	KX009699
MAD/GM2_5/12	KP792365	RSA_173_GD-CHN_2015	KX009686
RSVA/Homo_sapiens/USA/TH_10603/2013	KU950596	A/Shiraz/12/2016	KU716108
MON-3-88	Z33425	A/Shiraz/19/2016	KU716109

G gene nucleotide sequences for St. Petersburg RSV-A isolates were submitted in GenBank: MH142221–MH142238 and MK386440–MK386445.

## Data Availability

The data presented in this study are openly available in GenBank, reference number MH142221–MH142238 and MK386440–MK386445.

## Supplementary Materials

The following are available online at https://www.mdpi.com/1999-4915/13/1/119/s1. Figure S1: Genetic diversity of RSV sampled in different regions of the world (based on 1491 sequences available in the GenBank database). Genetic distance was calculated from the matrix containing squared root of the pairwase distances using dist.alignment function from “seqinr” package [22]. Black line in the box represents the median value, the length of the box represents the interquartile range (IQR), i.e. the difference between the lower (Q1) and the upper (Q3) quartile. Figure S2: G protein amino acid alignment of St. Petersburg RSV-A isolates, 2013–2014 epidemic season. With grey shadow for 2.1 subcluster, orange—3.1 subcluster. All substitutions discussed are filled with grey, 2.1 subcluster defining substitutions are filled with green, extra substitution defining 3.1 subcluster is filled with brown. Table S1: The occurrence of amino acid substitutions with predicted functional significance in RSV-A G protein dataset (CD—central domain, TMD—transmembrane domain, I HVR—I hypervariable region, II HVR—II hypervariable region). Unique substitutions observed in Russian sequences only are highlighted.
