# Supplementary material for: Respiratory Syncytial Virus G Protein Sequence Variability among Isolates from St. Petersburg, Russia, during the 2013–2014 Epidemic Season
Source: Viruses. 2021 Jan 17;13(1):119. doi: 10.3390/v13010119 (PMC7830914; doi:10.3390/v13010119)
Supplement: Supplementary file 1 [file viruses-13-00119-s001.zip › Supplementary figure 1.pdf]

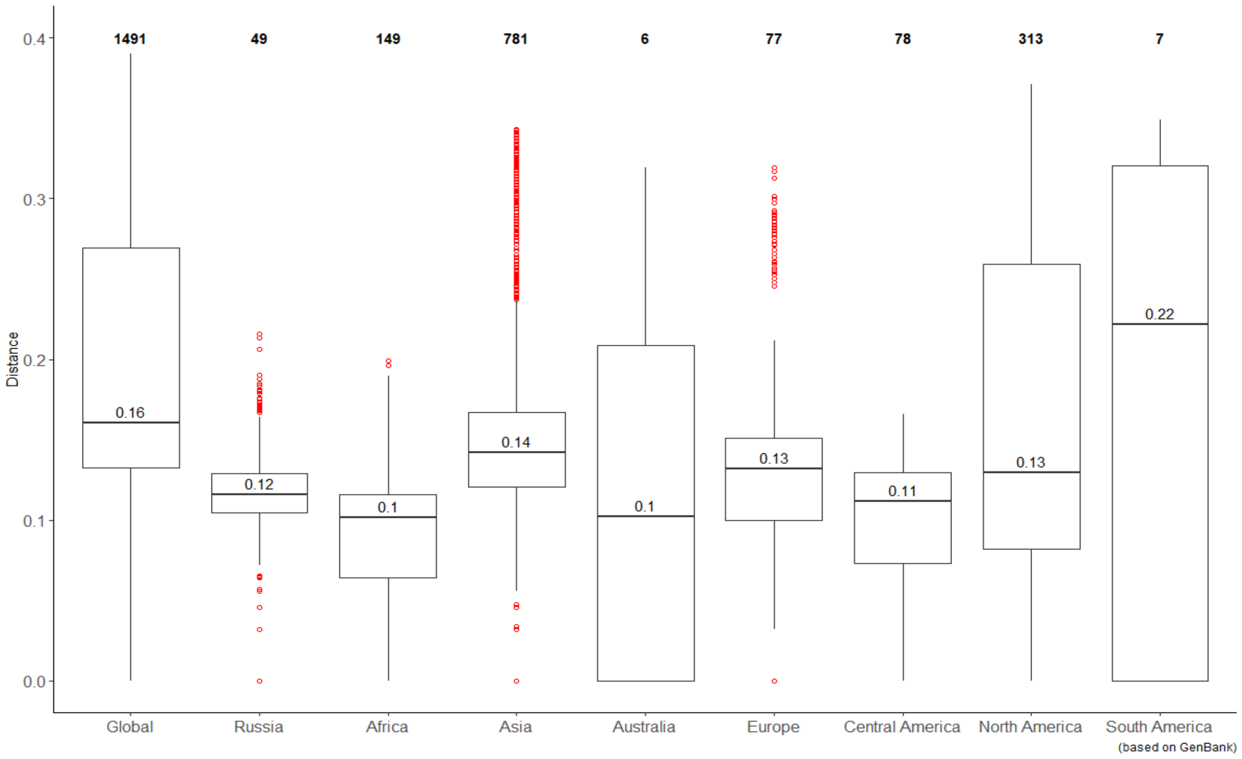

Supplementary figure 1. *Genetic diversity of RSV sampled in different regions of the world (based on 1491 sequences available in the GenBank database). Genetic distance was calculated from the matrix containing squared root of the pairwase distances using dist.alignment function from "seqinr" package [47]. Black line in the box represents the median value, the length of the box represents the interquartile range (IQR), i.e. the difference between the lower (Q1) and the upper (Q3) quartile*
