# Supplementary material for: Respiratory Syncytial Virus G Protein Sequence Variability among Isolates from St. Petersburg, Russia, during the 2013–2014 Epidemic Season
Source: Viruses. 2021 Jan 17;13(1):119. doi: 10.3390/v13010119 (PMC7830914; doi:10.3390/v13010119)
Supplement: Supplementary file 1 [file viruses-13-00119-s001.zip › Supplementary figure 2.pdf]

```
                                         10        20        30        40        50        60        70        80        90       100
                               ....|....|....|....|....|....|....|....|....|....|....|....|....|....|....|....|....|....|....|....|
ON67-1210A                     MSKTKDQRTAKTLERTWDTLNHLLFISSCLYKLNLKSIAQITLSILAMIISTSLIIAAIIFIASANHKVTLTTAIIQDATNQIKNTTPTYLTQNPQLGIS
Long                           ...N..........K.............G...........................T.......................S............D......
A2                             ...N.................................V................................P.........S...................
RSVA/St_Petersburg/479/2014    .........................................I..........................................................
RSVA/St_Petersburg/878/2014    ....................................................................................................
RSVA/St_Petersburg/1086/2014   ....................................................................................................
RSVA/St_Petersburg/1743/2014   ......P.......................................................................S.....................
RSVA/St_Petersburg/3513/2014   ....................................................................................................
RSVA/St_Petersburg/3546/2014   ___N...H............................................................................................
RSVA/St_Petersburg/4210/2014   ....................................................................................................
RSVA/St_Petersburg/5904/2014   ..............................................................................V.....................
RSVA/St_Petersburg/5934/2014   ......P.............................................................................................
RSVA/St_Petersburg/7108/2014   ...P.....................................I..........................................................
RSVA/St_Petersburg/10891/2014  ___.................................................................................................
RSVA/St_Petersburg/14959/2014  ....................................................................................................
RSVA/St_Petersburg/15849/2014  .......H............................................................................................
RSVA/St_Petersburg/16063/2014  ....Q...............................................................................................
RSVA/St_Petersburg/6021/2014   ___N.....................................I..........................................................
RSVA/St_Petersburg/7036/2014   ----H...............................................................................................
RSVA/St_Petersburg/5133/2014   -----------.........................................................................................
RSVA/St_Petersburg/6959/2014   ---------.R.........................................................................................
RSVA/St_Petersburg/7028/2014   ....................................................................................................
RSVA/St_Petersburg/8951/2014   -----------........................................................................................G
RSVA/St_Petersburg/14597/2014  ....................................................................................................
RSVA/St_Petersburg/6602/2014   ------..............................................................................................
RSVA/St_Petersburg/5577/2014   ___.................................................................................................
RSVA/St_Petersburg/11101/2014  ....................................................................................................
RSVA/St_Petersburg/13764/2014  ....................................................................................................
RSVA/St_Petersburg/5002/2014   ...........................................A........................................................
```

```
                                      110       120       130       140       150       160       170       180       190       200
                                ....|....|....|....|....|....|....|....|....|....|....|....|....|....|....|....|....|....|....|....|
ON67-1210A                      FSNLSGTTSQSTTILASTTPSAESTPQSTTVKIKNTTTTQILPSKPTTKQRQNKPQNKPNNDFHFEVFNFVPCSICSNNPTCWAICKRIPNKKPGKKTTT
Long                            .....EI...T.........GVK.NL.P....T.......TQ.............P............................................
A2                              P..P.EI...I.........GVK..L......T.......TQ.............PS...........................................
RSVA/St_Petersburg/479/2014     .................................I..................................................................
RSVA/St_Petersburg/878/2014     ....................................................................................................
RSVA/St_Petersburg/1086/2014    ....................................................................................................
RSVA/St_Petersburg/1743/2014    .................................I..................................................................
RSVA/St_Petersburg/3513/2014    ....................................................................................................
RSVA/St_Petersburg/3546/2014    .F..................................................................................................
RSVA/St_Petersburg/4210/2014    ...........................F........................................................................
RSVA/St_Petersburg/5904/2014    ...........I...............F........................................................................
RSVA/St_Petersburg/5934/2014    ....................................................................................................
RSVA/St_Petersburg/7108/2014    .................................I..................................................................
RSVA/St_Petersburg/10891/2014   ....................................................................................................
RSVA/St_Petersburg/14959/2014   .....R.....................F........................................................................
RSVA/St_Petersburg/15849/2014   .F..................................................................................................
RSVA/St_Petersburg/16063/2014   ....................................................................................................
RSVA/St_Petersburg/6021/2014    L................................I..................................................................
RSVA/St_Petersburg/7036/2014    .................................I...I..............................................................
RSVA/St_Petersburg/5133/2014    .F..................................................................................................
RSVA/St_Petersburg/6959/2014    ...........................F.......................................................S................
RSVA/St_Petersburg/7028/2014    .F..................................................................................................
RSVA/St_Petersburg/8951/2014    .F................................D.........E.......................................................
RSVA/St_Petersburg/14597/2014   ....................................................................................................
RSVA/St_Petersburg/6602/2014    ....................................................................................................
RSVA/St_Petersburg/5577/2014    ...........I........................................................................................
RSVA/St_Petersburg/11101/2014   ....................................................................................................
RSVA/St_Petersburg/13764/2014   ...........................F...................................................................M....
RSVA/St_Petersburg/5002/2014    .......................P............................................................................
```

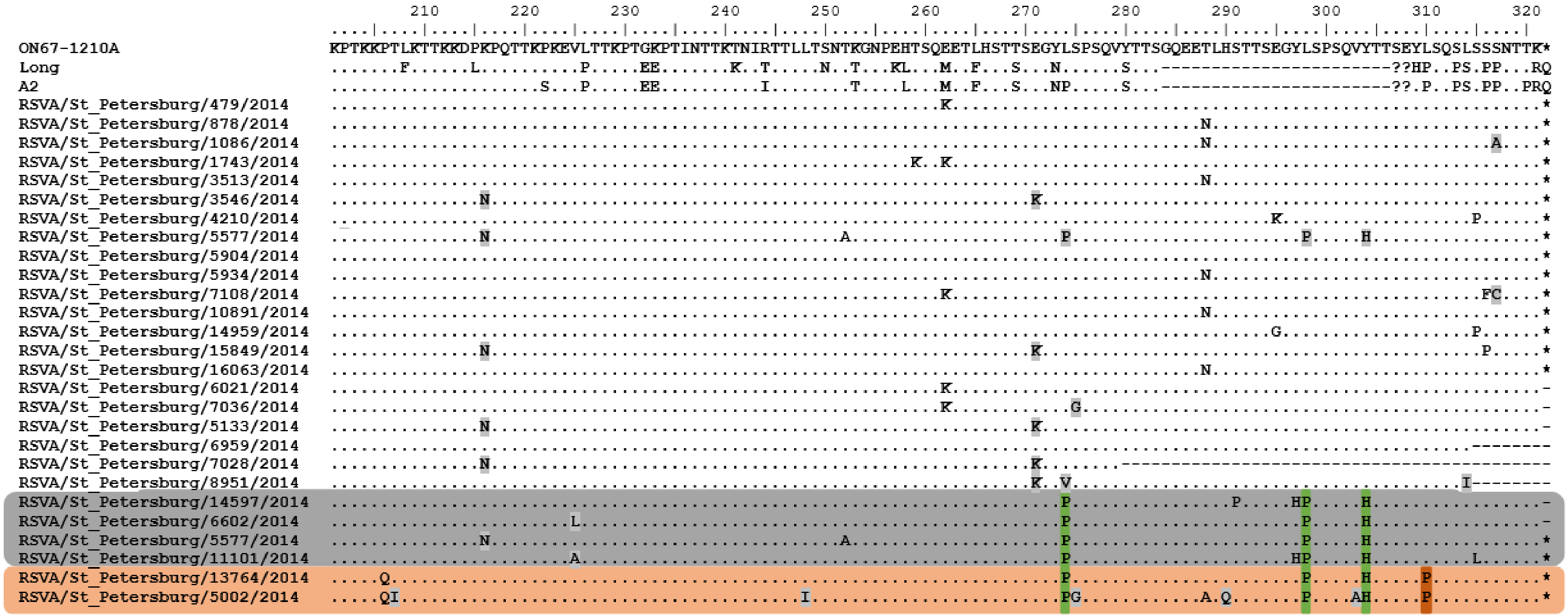


***Supplementary figure 2****. G protein amino acid alignment of St. Petersburg RSV-A isolates, 2013-2014 epidemic season. With grey shadow for 2.1 subcluster, orange – 3.1 subcluster. All substitutions discussed are filled with grey, 2.1 subcluster defining substitutions are filled with green, extra substitution defining 3.1 subcluster is filled with brown.*
