# Supplementary material for: Respiratory Syncytial Virus G Protein Sequence Variability among Isolates from St. Petersburg, Russia, during the 2013–2014 Epidemic Season
Source: Viruses. 2021 Jan 17;13(1):119. doi: 10.3390/v13010119 (PMC7830914; doi:10.3390/v13010119)
Supplement: Supplementary file 1 [file viruses-13-00119-s001.zip › Supplementary table 1.pdf]

Supplementary table 1. *The occurrence of amino acid substitutions with predicted functional significance in RSV-A G protein dataset (CD – central domain, TMD – transmembrane domain, I HVR – I hypervariable region, II HVR – II hypervariable region). Unique substitutions observed in Russian sequences only are highlighted.*

	Position																									
	CD					TMD		I HVR	CCR		II HVR (N-end)						II HVR (C-end)									
AA residue	4	5	7	8	11	42	44	102	177	184	196	206	207	216	225	248	271	274	275	290	298	303	304	310	314	317
?	1	0	0	0	5	0	0	0	0	0	0	0	0	0	0	0	0	0	0	2	0	0	0	1	4	3
A	1	0	0	0	0	3	1	0	0	1436	0	0	0	0	343	0	0	0	0	0	0	21	0	0	0	1
C	0	0	0	1	0	0	0	0	1	0	0	0	0	0	0	0	0	0	0	0	0	0	0	0	0	1
D	0	0	0	0	0	0	0	0	0	0	0	0	0	0	0	0	2	0	0	0	0	0	0	0	0	0
E	0	0	0	0	2	0	0	0	0	0	1	0	0	0	0	0	544	0	0	0	0	0	0	0	0	0
F	0	0	0	0	0	0	0	280	0	0	0	0	0	0	0	3	0	0	0	0	0	1	0	0	0	19
G	0	0	0	0	0	0	0	0	0	0	0	0	0	0	0	0	0	0	3	0	0	0	0	0	0	0
H	0	1	0	6	0	0	0	0	0	0	0	0	0	0	0	0	0	0	0	1417	0	0	272	0	0	0
I	0	0	0	0	0	9	0	0	0	0	0	0	36	17	1	23	0	0	0	0	0	250	0	2	1	0
K	0	1383	0	0	1402	0	0	0	0	0	1438	0	0	1395	0	0	21	0	0	0	0	0	0	0	0	0
L	0	0	0	0	0	0	0	0	0	0	0	1	0	0	20	1413	0	259	0	0	690	0	0	1312	548	0
M	0	0	0	0	0	0	0	0	0	0	1	0	0	0	0	0	0	0	0	0	0	0	0	0	0	0
N	19	0	0	0	1	0	0	0	1	0	0	0	0	23	0	0	0	0	1	4	0	0	0	0	0	0
P	2	0	2	0	0	0	0	0	0	1	0	1306	1	0	0	1	0	291	0	0	487	0	0	122	873	7
Q	0	1	1385	0	0	0	0	0	0	0	0	133	0	2	0	0	0	0	0	2	7	0	0	0	0	0
R	0	1	1	1381	2	0	0	0	1	0	0	0	0	2	0	0	0	0	0	5	0	0	0	0	0	0
S	0	0	0	0	0	0	1413	1156	1437	1	0	0	0	0	0	0	0	16	563	0	1	0	7	0	9	1401
T	1363	0	0	0	0	1402	0	4	0	2	0	0	1403	0	5	0	0	0	0	0	254	0	0	0	0	0
V	0	0	0	0	0	0	0	0	0	0	0	0	0	0	1071	0	0	1	0	0	0	1166	0	0	0	0
Y	0	0	0	0	0	0	0	0	0	0	0	0	0	1	0	0	0	0	0	10	0	0	1159	0	0	0
